# Hydrogen Sulfide-Releasing Carbonic Anhydrase Inhibitors Effectively Suppress Cancer Cell Growth

**DOI:** 10.3390/ijms251810006

**Published:** 2024-09-17

**Authors:** Alessandro Bonardi, Alessio Nocentini, Viviana de Luca, Clemente Capasso, Eslam B. Elkaeed, Wagdy M. Eldehna, Claudiu T. Supuran

**Affiliations:** 1 Department of NEUROFARBA, Pharmaceutical and Nutraceutical Section, University of Firenze, Via Ugo Schiff 6, Sesto Fiorentino, 50019 Florence, Italy; claudiu.supuran@unifi.it; 2 Department of Biology, Agriculture and Food Sciences, National Research Council (CNR), Institute of Biosciences and Bioresources, 80131 Naples, Italy; viviana.deluca@ibbr.cnr.it (V.d.L.); clemente.capasso@ibbr.cnr.it (C.C.); 3 Department of Pharmaceutical Organic Chemistry, Faculty of Pharmacy (Boys), Al-Azhar University, Cairo 11884, Egypt; eslamkaeed@azhar.edu.eg; 4 Department of Pharmaceutical Chemistry, Faculty of Pharmacy, Pharos University in Alexandria, Canal El Mahmoudia St., Alexandria 21648, Egypt; wagdy2000@gmail.com; 5 Department of Pharmaceutical Chemistry, Faculty of Pharmacy, Kafrelsheikh University, Kafrelsheikh 33516, Egypt

**Keywords:** hydrogen sulfide, donor, carbonic anhydrase, inhibitor, hybrid, multitarget, tumor, cancer, antitumor, in vitro

## Abstract

This study proposes a novel therapeutic strategy for cancer management by combining the antitumor effects of hydrogen sulfide (H_2_S) and inhibition of carbonic anhydrases (CAs; EC 4.2.1.1), specifically isoforms IV, IX, and XII. H_2_S has demonstrated cytotoxicity against various cancers at high concentrations. The inhibition of tumor-associated CAs leads to lethal intracellular alkalinization and acidification of the extracellular tumor microenvironment and restores tumor responsiveness to the immune system, chemotherapy, and radiotherapy. The study proposes H_2_S donor–CA inhibitor (CAI) hybrids for tumor management. These compounds effectively inhibit the target CAs, release H_2_S consistently, and exhibit potent antitumor effects against MDA-MB-231, HCT-116, and A549 cancer cell lines. Notably, some compounds display high cytotoxicity across all investigated cell lines. Derivative **30** shows a 2-fold increase in cytotoxicity (0.93 ± 0.02 µM) under chemically induced hypoxia in HCT-116 cells. These compounds also disturb the cell cycle, leading to a reduction in cell populations in G_0_/G_1_ and S phases, with a notable increase in G_2_/M and Sub-G_1_. This disruption is correlated with induced apoptosis, with fold increases of 37.2, 24.5, and 32.9 against HCT-116 cells and 14.2, 13.1, and 19.9 against A549 cells compared to untreated cells. These findings suggest the potential of H_2_S releaser–CAI hybrids as effective and versatile tools in cancer treatment.

## 1. Introduction

Hypoxia is strongly correlated with tumor growth, malignant progression, non-responsiveness to the immune system, and resistance to chemotherapy and radiotherapy [1,2,3,4]. An oxygen-deprived environment is the primary trigger for the overexpression of Hypoxia-Inducible Factor-1 (HIF-1) in tumors, while uncontrolled tumor growth causes the initiation of the Warburg effect [1,2,3,4,5,6,7]. The Warburg effect consists of a pivotal metabolic transformation within normoxic and hypoxic cancer cells, amplifying anaerobic glycolysis by 100–200 times [5,6,7]. This heightened glycolytic activity results in the massive conversion of pyruvate into lactate, facilitating ATP production at a rate 10–100 times faster than the complete oxidation of glucose [5,6,7]. Indeed, in normoxic glycolytic tumors, only about 5% of the entire produced pyruvate is oxidized in the Krebs cycle. Furthermore, the overproduced pyruvate and lactate are utilized by the tumors for trophic purposes in the biosynthesis of amino acids, nucleotides, and lipids [5,6,7,8].

**Figure 1:**
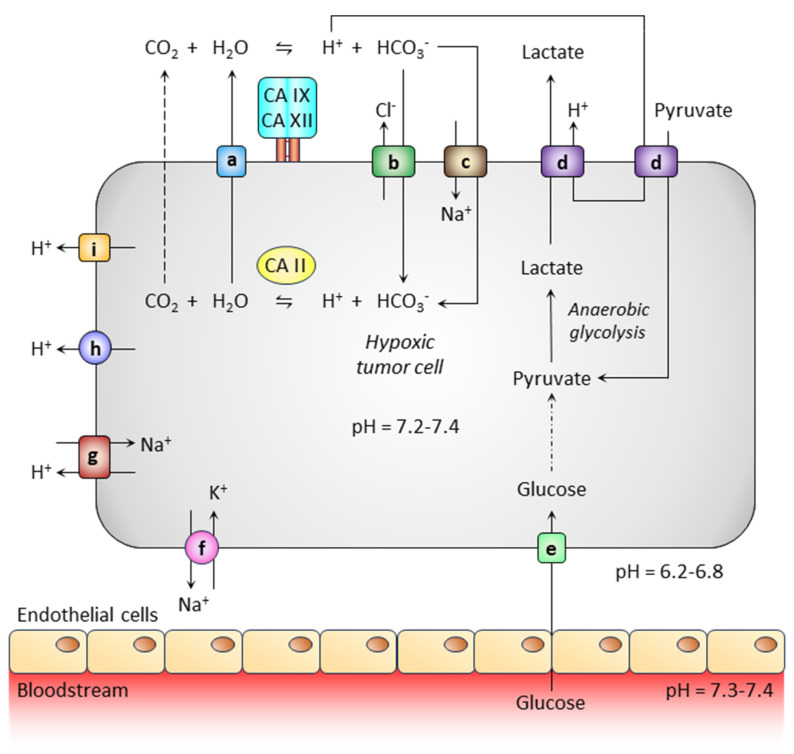
Schematic representation of a hypoxic tumor cell and proteins/processes implicated in its pH regulation. The transmembrane CA IX, XII, and CA IV (omitted), with an extracellular active site, catalyze the CO_2_ hydration to bicarbonate and proton. Other involved proteins include (a) aquaporins, (b) anion exchangers (AE1–AE3 isoforms), (c) Na^+^/HCO_3_^−^ cotransporters, (d) mono-carboxylate carrier (MTC1 and MTC4), (e) glucose transporter (GLUT1 and GLUT3), (f) ATP-dependent Na^+^/K^+^ antiporter, (g) Na^+^/H^+^ antiporter, (h) plasma membrane proton pump H^+^/ATPase, and (i) H^+^ channels.

To adapt cancer cells to hypoxic conditions and acidosis, resulting from the fermentative metabolism of the Warburg effect, HIF-1 induces the upregulation of multiple genes, including those responsible for the overexpression of glucose (GLUT1 and GLUT3) and proton transporters, lactate-proton symporters (MCT1 and MTC4), angiogenetic factors (e.g., vascular endothelial growth factor, VEGF), and the tumor-associated isoforms IX and XII of carbonic anhydrases (CA, EC 4.2.1.1) [8,9,10]. These CAs play a key role in catalyzing the reversible hydration of carbon dioxide (CO_2_) into bicarbonate (HCO_3_^−^) and a proton (H^+^), contributing to the cellular response mechanism (Figure 1) [11,12]. The combination of several proton export mechanisms, with inadequate vascular drainage and CAs activity, upholds an intracellular pH in the range of 7.2–7.4, leading to an extracellular pH of 6.2–6.8 [2,10,11,12]. The acidification of the tumoral microenvironment is strongly linked to tumor propagation, malignant progression, non-responsiveness to the immune system, and resistance to chemotherapy and radiotherapy [1,2,8,10,11,12,13,14,15]. Moreover, the tumor-associated CAs sustain the cancer growth by refurnishing the carbamoyl phosphate II and N5-carboxyaminoimidazole ribonucleotide synthase enzymes of HCO_3_^−^ in the de novo synthesis of nitrogenous bases [10].

Specifically, the expression of CA IX and XII is significantly upregulated across many types of tumors [2,16,17,18,19,20,21,22,23,24,25] and is regulated downward by the wild-type von Hippel–Lindau tumor suppressor protein (pVHL) [26,27,28]. In certain cancer cells, mutations in the VHL gene lead to a pronounced upregulation of tumor-associated CA isoforms due to persistent activation of HIF-1 [29,30]. Recently, the isoform hCA IV was shown to be primarily localized on the membrane of astrocytes, playing a crucial role in interstitial pH regulation [31] and the control of monocarboxylate transport in brain cells [32,33,34,35] along with CA IX and XII [36,37,38]. Although anticancer application due to hCA IV inhibition is not yet clearly established, several studies have demonstrated that CA IX and XII are promising targets for the development of carbonic anhydrase inhibitors (CAIs) as innovative anticancer drugs [39,40,41]. Presently, the benzenesulfonamide inhibitor SLC-0111, selective against CA IX and XII, is undergoing phase II clinical trials as a potential antitumor agent specifically designed for the management of metastatic solid hypoxic tumors [42,43,44]. 

Hydrogen sulfide (H_2_S), alongside nitric oxide (NO) and carbon monoxide (CO), constitutes the third gasotransmitter in the human body and has been extensively explored for biomedical applications over the past three decades [45]. Three enzymes with distinct subcellular and tissue distributions, known as cystathionine-β-synthase (CBS), cystathionine-γ-lyase (CSE), and 3-mercaptopyruvate sulfurtransferase (3-MST), are responsible for the endogenous synthesis of H_2_S, using *l*-cysteine, *l*-homocysteine, and 3-mercaptopyruvate as substrates, respectively [45,46,47,48]. Given the absence of a specific receptor or signaling pathway, hydrogen sulfide exerts its effects in a cell/tissue/species-dependent manner through paracrine action regulating various crucial physiological processes [46,49]. Among these, H_2_S modulates the vascular tone and blood pressure, various leukocytic functions, angiogenesis, cardiac function, neurotransmission and nociception, penile erectile, ATP synthesis, and scavenger action [50,51,52,53]. 

Currently, a dose-dependent impact of this gasotransmitter is evident in cancer [54]. At lower concentrations, hydrogen sulfide fosters cancer progression, induces angiogenesis (e.g., VEGF overexpression), and inhibits tumor cell apoptosis [55]. The latter effect is attributed to its scavenger role against reactive oxygen species (ROS), mitigating DNA damage associated with mitochondrial disruption [55]. Moreover, optimal H_2_S levels expedite the cancer cell cycle by modulating the phosphorylation level of protein kinase B (PKB or Akt) and enhancing ATP synthesis through sulfhydration of ATP synthase (Complex V) [55].

Conversely, an elevated concentration of exogenous H_2_S elucidates a cytotoxic effect on cancer cells. This manifests through the promotion of cytosolic migration of cytochrome C, uncontrolled intracellular acidification, induction of oxidative stress, interruption of ATP synthesis, cessation of cell replication in the G_2_ and M phases, downregulation of antiapoptotic genes, and acceleration of apoptosis [54,56].

**Figure 2:**
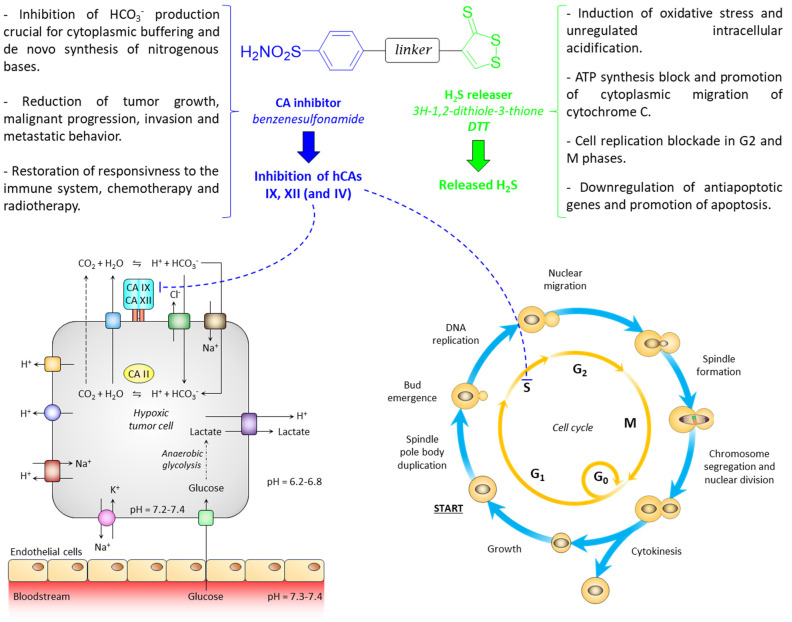
Rational design of H_2_S releaser–CAI hybrid derivatives as antitumor agents.

In this study, we propose for the first time the application of hydrogen sulfide donor-CAI derivatives as antitumor agents, combining the action of exogenous H_2_S and inhibition of the tumor-associated CA isoforms for the management of cancer (Figure 2).

## 2. Results and Discussion

### 2.1. Drug Design and Chemistry

The development rationale for hydrogen sulfide donor-CAI derivatives takes into account four crucial aspects: (i) the antitumor activity associated with CA inhibition, (ii) the retained CA inhibition by the hybrid post-H_2_S release, (iii) the cytotoxic impact of certain H_2_S concentrations on cancer cells, and iv) the awareness of hydrogen sulfide toxicity for the selection of the appropriate H_2_S-releaser scaffold.

In particular, the inhibition of the overexpressed CA IX and XII prevents tumor cell acclimatization to hypoxic conditions and the Warburg effect, blocking intracellular pH alkalinization and extracellular environment acidification, triggered to counteract the metabolic acidosis [2,10]. Consequently, tumor growth is halted, both due to the progressive inactivation of metabolic enzymes—caused by acidosis—and the lack of the necessary bicarbonate for de novo synthesis of nitrogenous bases, crucial in the S phase of the cell cycle in hypoxia, and other metabolic reactions (e.g., lipogenesis) [2,10]. Moreover, by restoring the correct extracellular pH, the CA inhibition repristinates the responsiveness to the immune system, chemotherapy, and radiotherapy of the tumors, silencing the malignant progression, invasion, and metastasis behavior [2,10,43,44].

It is crucial to underscore that a hypoxic tumor comprises a heterogeneous mass with cells in different hypoxia states [1,2,3,4,5,6,7,8]. Thus, the respiratory chain and oxidative phosphorylation are minimally active in hypoxic tumor cells located near the bloodstream, where oxygen availability is limited but present [1,2,3,4,5,6,7,8]. Exogenous hydrogen sulfide entirely disrupts oxygen-dependent metabolism in these cells, inducing apoptosis throughout the entire tumor [55]. Again, the H_2_S cytotoxic mechanism could make effective the designed hybrids also against normoxic glycolytic tumors sensitive to the gasotransmitter, which normally express the tumor-associated CAs on the external membrane of the cancer cells, and non-glycolytic tumors [55,57].

Furthermore, the development of H_2_S donor–CAI hybrids carefully addressed the potential toxicity of H_2_S, a recognized harmful gas. Inhalation of elevated concentrations (>20 ppm) may result in its absorption into the bloodstream and tissues, leading to obstruction of the cell respiratory chain through binding to the iron in cytochrome C oxidase. This cascade effect induces oxidative stress and ATP synthesis inhibition, posing a potential fatality risk (ppm = 1000). Consequently, meticulous regulation of H_2_S release is pivotal for achieving an optimum therapeutic concentration [48,54].

The herein re-purposed drug design study initiates with the enzyme-activated 4-substituted 3H-1,2-dithiole-3-thione chemotype (**DTT**, Figure 2), selected for its specific therapeutic action and the requisite rate of H_2_S release, measured and validated in our previous work [58]. In selecting CAI scaffolds for incorporation into the molecular hybrids, we opted for the benzenesulfonamide chemotype, used for the design of SLC-0111. This decision was driven by its exceptional hCA inhibition potency and demonstrated suitability for drug design strategies focused on enhancing isoform selectivity [41].

**Scheme 1:**
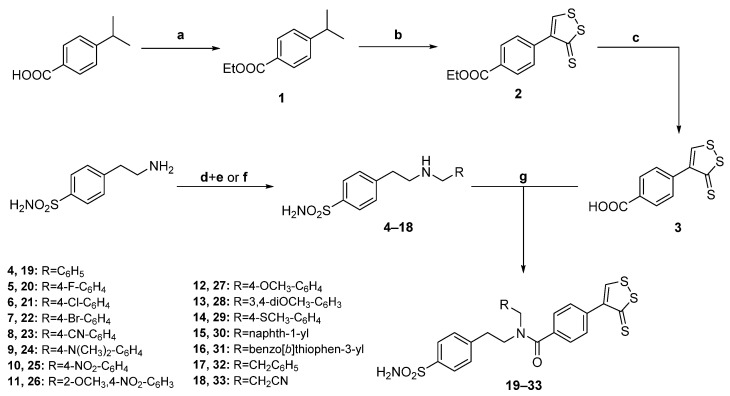
Synthesis of H_2_S releaser-CAI derivatives **19**–**33**. Reagents and conditions: (a) SOCl_2_, dry EtOH, 0 → 60 °C, 6 h; (b) S_8_, 135 → 220 °C, 6–8 h; (c) H_2_SO_4_ 9M, CH_3_COOH, 100 °C, 4 h; (d) RCHO, dry MeOH, reflux, 4 h; (e) NaBH_4_, dry MeOH, reflux, 0.5–2 h; (f) R-X, dry DMF, r.t or 60 °C, 0.5–6 h; (g) DIPEA, PyBOP, dry DMF, r.t, o.n. [58].

The H_2_S releaser-CAIs **19**–**33** were reported previously as anti-inflammatory agents with great efficacy in the management of arthritis in vivo [58]. Their synthesis starts with the preparation of the **DTT** core, incorporating a p-COOH-phenyl moiety at position 4 for subsequent hybridization with the CAI scaffold (Scheme 1). In brief, the protection of 4-isopropylbenzoic acid as ethyl ester **1** occurs using SOCl_2_ in dry ethanol, and a cyclization to form **DTT** intermediate **2** is achieved in molten sulfur at 220 °C. Again, the ester group is hydrolyzed with H_2_SO_4_ in acetic acid at 100 °C, yielding carboxylic acid **3**.

To prevent undesired attack on the thionic group of the **DTT** scaffold with primary amines, forming imine-like derivatives even at very low temperatures, secondary amines of 4-(2-aminoethyl)benzenesulfonamide **4**–**18** were prepared by reductive amination with the appropriate aromatic aldehydes and sodium borohydride in dry methanol or nucleophilic substitution with the proper halides in dry DMF. Finally, the CAI scaffolds and **DTT** (**3**) were coupled in dry DMF at room temperature, using PyBOP and DIPEA to attain hybrids **19**–**33**.

### 2.2. Carbonic Anhydrase Inhibition

Extensive structure–activity relationship (SAR) studies have been previously discussed [58]. Here is a summary of the inhibition profile of hybrids **19**–**33** against hCA isoforms I, II, IV, IX, and XII evaluated by a stopped-flow kinetic assay using acetazolamide (AAZ) as the standard inhibitor (Table 1) [59].

Table 1 provides the inhibition constants (K_I_s) for compounds **19**–**33** against the examined hCAs. In particular, cytosolic hCA I and II are considered off-target isoforms due to their ubiquity and association with side effects in anticancer treatment [10]. In contrast, hCAs IV, IX, and XII are target isoforms as they are overexpressed in hypoxic and cerebral tumors [39,40,60].

(i) Most compounds showed weak inhibition against hCA I, with K_I_ values in the micromolar range, except for derivatives **19** and **28**, which inhibited hCA I in the high nanomolar range. All compounds exhibited more pronounced hCA II inhibition compared to hCA I, except for compound **29**.
(ii) Derivatives **19**, **20**, **27**, **28**, and **32** inhibited hCA II in the medium–high nanomolar range. The elongation of the N-substituent from benzyl (**19**) to phenethyl (**32**) reduced inhibitory action against hCA I and hCA II.
(iii) Tertiary amide derivative **33**, without an aromatic scaffold on the N-substituent, showed the highest activity against hCAs I and II. The reduced steric hindrance of **33** likely favors ligand binding in the narrow active sites of these isoforms.
(iv) Regarding hCA IV, only derivatives **23** and **31** exhibited K_I_ values below 100 nM, making them the most potent and selective against hCA IV over hCA II. Other compounds inhibited hCA IV in the high nanomolar to low micromolar range.
(v) hCA IX and XII were the most inhibited isoforms, with K_I_s in the low nanomolar range. Compounds **31** and **26** were the most potent and selective hCA IX inhibitors. The elongation of the N-substituent from **19** to **32** led to a K_I_ exceeding 100 nM. The remaining compounds demonstrated hCA IX inhibition within the medium nanomolar range. For hCA XII, several compounds showed single-digit K_I_s. Derivatives **30** and **26** were the most potent hCA XII inhibitors, while **29** and **30** were the most selective over hCA II.

In summary, coupling bulky secondary amine benzenesulfonamide derivatives to the H_2_S donor nucleus was a successful strategy for developing potent hCA IX and XII inhibitors with high selectivity over off-target hCAs. The incorporation of N-alkyl aromatic substituents directed the designed H_2_S releaser-CAI derivatives to preferentially bind to the larger active sites of hCA IX and XII. The 2-ethylcyano derivative **33**, with the least steric encumbrance, exhibited a significant loss of target/off-target selectivity.

### 2.3. Evaluation of H_2_S Release

We evaluated the H_2_S-releasing properties of the most potent and selective compounds (**22**, **23**, **26**, **29**, and **30**) against isoforms IV, IX, and XII using a spectrophotometric methylene blue (MB^+^) method [61,62]. Our previous work outlined the H_2_S release profiles of compounds **26**, **30**, **31**, and the 4-substituted-3H-1,2-dithiole-3-thione precursor **3** at 150 µM. H_2_S was liberated, captured by zinc acetate to form zinc sulfide, and reacted with N,N-dimethyl-1,4-phenylenediamine in the presence of Fe^3+^ to produce methylene blue, measurable at 667 nm (MB^+^ form) or 748 nm (MBH^2+^) [58,63,64]. The H_2_S release was monitored over time and quantified using a Na_2_S calibration curve (1–300 µM) [58]. Incubating compound **3** and its derivatives in phosphate buffer at 37 °C, with or without thiol compounds (l-cysteine or glutathione), resulted in minimal H_2_S release. Therefore, we explored alternative triggering mechanisms, including enzymatic/metabolic triggers in rat tissue homogenates [65,66,67].

**Figure 3:**
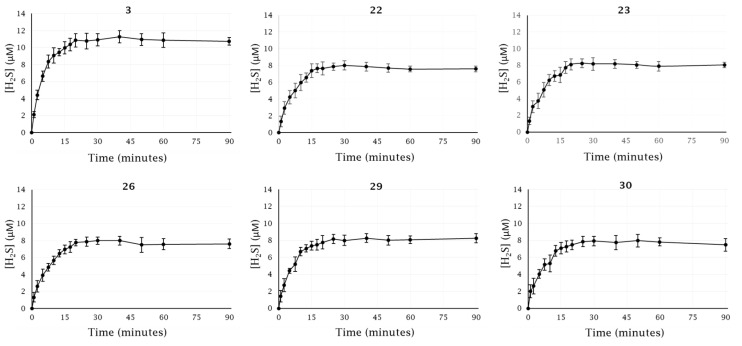
H_2_S-releasing profile of compounds **3**, **22**, **23**, **26**, **29**, and **30** (150 µM). Compounds were incubated in rat liver homogenate at 37 °C. H_2_S concentration was determined spectrophotometrically at timed intervals. Data are expressed as mean ± SEM of three independent assays.

Incubation of compounds **22**, **23**, **26**, **29**, and **30** at 37 °C in rat liver homogenate led to an immediate and sustained H_2_S release over 90 min, suggesting a metabolic activation event. Table 2 details the C_max_ and t/2 for the tested compounds. Compound **3** exhibited the highest H_2_S release rate and C_max_, peaking at 12.1 µM after 20 min with a t/2 of 3.1 min. In contrast, amide derivatives **22**, **23**, **26**, **29**, and **30** showed reduced H_2_S release, with C_max_ values ranging from 7.9 to 8.2 µM and t/2 values between 4.6 and 5.2 min (Figure 3).

### 2.4. Anticancer Activity

#### 2.4.1. In Vitro Antiproliferative Studies

An in vitro investigation assessed the antiproliferative activity of the most potent and selective compounds (**22**, **23**, **26**, **29**, and **30**) against three different cancer cell lines: MDA-MB-231 (triple-negative breast cancer), HCT-116 (colon cancer), and A549 (lung cancer).

The three in-house cell lines (breast, colon, and lung) were chosen based on two primary factors: (a) Responsiveness to H_2_S. There is well-documented evidence in the literature demonstrating these cell lines’ responsiveness to hydrogen sulfide-releasing agents [68,69,70,71,72,73,74,75,76]. (b) CA Expression. These cell lines are known to express carbonic anhydrase IX and/or XII [74,75,76,77], making them suitable for studying the effects of CA inhibition.

By selecting cell lines that meet both criteria, we aimed to create a robust experimental model for evaluating our proposed therapeutic strategy. This approach allows us to investigate the combined effects of H_2_S release and CA inhibition in relevant cancer types.

The MTT assay results are expressed as IC_50_ (μM) values, utilizing Staurosporine as the reference cytotoxic drug.

In this screening, under normoxic conditions (Table 3), the tested compounds generally demonstrated the highest antiproliferative activity against HCT-116 (IC_50_ = 1.24 ± 0.04 − 10.13 ± 0.66 μM), followed by MDA-MB-231 (IC_50_ = 1.53 ± 0.04 − 10.43 ± 0.61 μM) and A549 (IC_50_ = 1.78 ± 0.04 − 60.91 ± 2.17 μM). In particular, the majority of compounds exhibited superior cytotoxic activity compared to the standard Staurosporine, with IC_50_ values ≤ 10 μM, except for compounds **22** and **29** against A549. Among the most potent compounds (**23**, **26**, and **30**), derivative **23** stood out for its superior cytotoxicity towards all investigated cell lines.

Furthermore, the cytotoxic activity on the most inhibited cell line, HCT-116, was also evaluated under hypoxic conditions induced by cobalt(II) chloride hexahydrate (Table 4). Notably, derivative **30** exhibited stronger cytotoxicity, with a 2-fold increase compared to normoxia, followed by **26** and **23**, confirming an activity attributed to tumor-associated CA inhibition. It is important to note that a general decline in the cytotoxicity of compounds and Staurosporine is likely due to the chemoresistance acquired by the tumor cells in hypoxia. Changes in the microenvironment pH may interfere with the absorption or alter the correct ionization state of the compounds. Despite that, derivative **30** passes from 5-fold to 10-fold increase activity concerning Staurosporine from normoxic to hypoxic conditions.

#### 2.4.2. Cell Cycle Analysis

**Figure 4:**
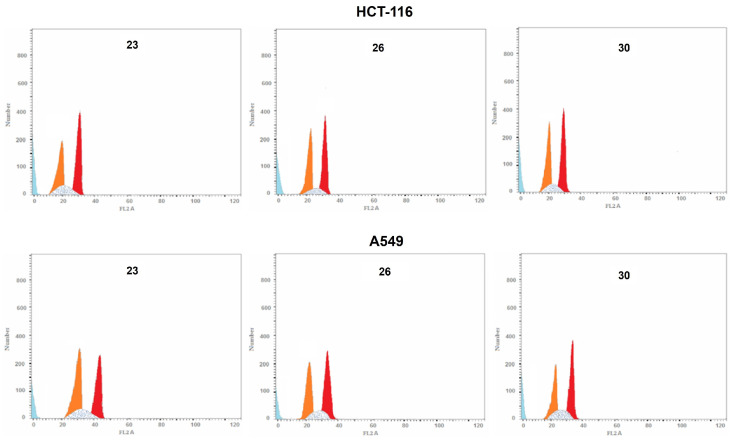
Effects of compounds **23**, **26**, and **30** on the cell cycle phases of HCT-116 and A549 cancer cells.

The ability of the most cytotoxic compounds, specifically **23**, **26**, and **30**, to disrupt the cell cycle of the most inhibited cell lines (HCT-116 and A549) was assessed using a DNA flow cytometric analysis (Figure 4 and Table 5). Untreated cells were employed as the control. The incubation of HCT-116 and A549 cells for 24 h with the tested compounds at their IC_50_ concentrations (Table 3) affected the progression of the cell cycle. Indeed, while there was a decrement in the cell percentage in G_0_/G_1_ and S phases in comparison to control cells, a significant increment in the cell population in G_2_/M and Sub-G_1_ was observed (Table 5).

#### 2.4.3. Annexin V-FITC/PI Dual Staining Assay

**Figure 5:**
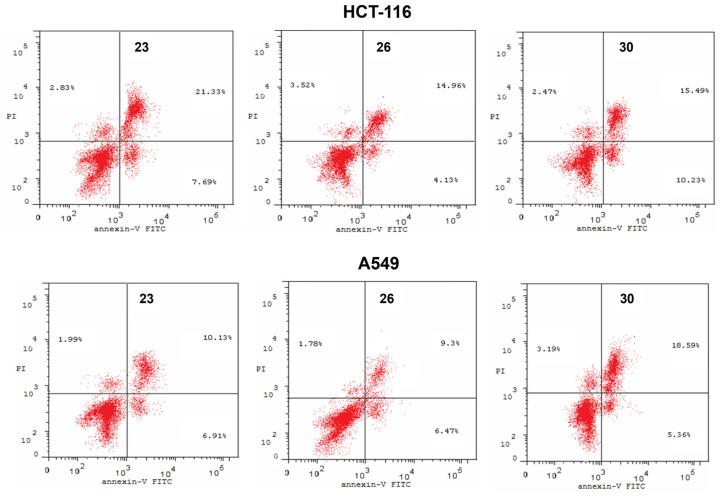
Effects of compounds **23**, **26**, and **30** on the percentage of annexin V-FITC-positive staining in HCT-116 and A549 cancer cells. The experiments were conducted in triplicates. The four quadrants are identified as LL, viable; LR, early apoptotic; UR, late apoptotic; UL, necrotic.

To ascertain whether the growth-inhibitory effects of compounds **23**, **26**, and **30** correlate with apoptosis induction as suggested by the heightened sub-G_1_ population in treated HCT-116 and A549 cells (Figure 4 and Table 5), an Annexin V-FITC/PI double staining (AV/PI) apoptosis assay was conducted (Figure 5 and Table 6). The AV-FITC/PI assay results revealed that the treatment of HCT-116 and A549 cells with hybrids **23**, **26**, and **30** induced apoptosis, as evidenced by a significant increase in the percentage of apoptotic cells in both the early and late apoptosis phases. In particular, a significant overall increase in apoptosis compared to the untreated control was noted for derivatives **23**, **26**, and **30**, with respective fold increases of 37.2, 24.5, and 32.9 against HCT-116 cells, and 14.2, 13.1, and 19.9 against A549 cells (Figure 5 and Table 6).

## 3. Materials and Methods

### 3.1. Chemistry

Anhydrous solvents and all reagents were purchased from Merck, Fluorochem, and TCI. All reactions involving air- or moisture-sensitive compounds were performed under a nitrogen atmosphere using dry glassware and syringe techniques to transfer solutions. Nuclear magnetic resonance (1H-NMR) spectra were recorded using a Bruker Advance III 400 MHz spectrometer (Bruker, Billerica, MA, USA) in DMSO-d6. Chemical shifts are reported in parts per million (ppm), and the coupling constants (J) are expressed in Hertz (Hz). Splitting patterns are designated as follows: s, singlet; d, doublet; t, triplet; q, quadruplet; m, multiplet; dd, doublet of doublets. The assignment of exchangeable protons was confirmed by the addition of D_2_O. Analytical thin-layer chromatography (TLC) was carried out on Sigma-Aldrich silica gel F-254 plates. Flash chromatography purifications were performed on Sigma-Aldrich Silica gel 60 (230-400 mesh ASTM) as the stationary phase, and ethyl acetate/n-hexane or MeOH/DCM were used as eluents. Melting points (mps) were measured in open capillary tubes with a Gallenkamp MPD350.BM3.5 apparatus (Gallenkamp and Company Limited, Cambridge, UK) and were uncorrected. HRMS analyses were performed on a Bruker Daltonics MicrOTOF-Q II mass spectrometer (Bruker, Billerica, MA, USA). All spectra were in accord with the assigned structures. All hybrid compounds were >95% pure. The purity of target compounds was assessed by HPLC using an Agilent 1200 Series gradient HPLC system (Agilent Technologies, Santa Clara, CA, USA) with a Luna PFP column (3 μm, 2 mm × 30 mm) at a flow rate of 0.25 mL/min and a linear gradient of the mobile phase, i.e., 10 mM formic acid and 5 mM ammonium formate in mQ water solution (solvent A) and 10 mM formic acid and 5 mM ammonium formate in methanol (solvent B). Synthetic procedures and compound characterization are reported in ref. [58].

### 3.2. Carbonic Anhydrase Inhibition Assay and KI Determination

An Applied Photophysics stopped-flow instrument (Applied Photophysics Ltd, Leatherhead, UK) was used for assaying the CA-catalyzed CO_2_ hydration activity [59]. Phenol red (at a concentration of 0.2 mM) was used as an indicator, working at the absorbance maximum of 557 nm, with 20 mM Hepes (pH 7.5) as a buffer and 20 mM Na_2_SO_4_ for maintaining constant the ionic strength, following the initial rates of the CA-catalyzed CO_2_ hydration reaction for a period of 10−100 s. The CO_2_ concentrations ranged from 1.7 to 17 mM for the determination of the kinetic parameters and inhibition constants. For each inhibitor, at least six traces of the initial 5−10% of the reaction were used for determining the initial velocity. The uncatalyzed rates were determined in the same manner and subtracted from the total observed rates. Stock solutions of inhibitor (0.1 mM) were prepared in distilled−deionized water and dilutions up to 0.01 nM were made thereafter with the assay buffer. Inhibitor and enzyme solutions were preincubated together for 15 min at room temperature before the assay to allow for the formation of the E−I complex. The inhibition constants were obtained by nonlinear least-squares methods using Prism 7 (GraphPad Software, La Jolla, CA, USA) and the Cheng−Prusoff equation and represent the mean of at least three different determinations. The enzyme concentrations were in the range of 3–11 nM. All hCA isoforms were recombinant ones obtained in-house [58].

### 3.3. Spectrophotometric Evaluation of H_2_S Release

Rat liver was homogenized in an ice-cold 50 mM potassium phosphate buffer (pH 7.4). An optimal weight/volume ratio of 1/10 was determined from preliminary experiments. The 3*H*-1,2-dithiole-3-thione derivatives were prepared as a 20 mM solution in DMSO and incubated at 150 μM in phosphate buffer, either in the presence or absence of a thiol derivative (such as *l*-cysteine and glutathione) or in freshly diluted rat liver homogenate in airtight vials at 37 °C for various time intervals (0–90 min). The released H_2_S concentration was measured as described previously. Briefly, after incubation, zinc acetate (1 mM final concentration) was added to trap the released H_2_S, followed by trichloroacetic acid (10% *w*/*v*) to precipitate proteins and stop the H_2_S release reaction. Baseline H_2_S concentration was determined by adding trichloroacetic acid directly to the tissue homogenate without the compound. Then, *N*,*N*-dimethyl-*p*-phenylenediamine sulfate in 5M HCl (10 mM final concentration), and FeCl_3_ in 1.2M HCl (2 mM final concentration) were added to the mixture. The absorbance of the formed methylene blue was measured after 30 min at 25 °C at 748 nm (the maximum absorption peak of the MBH^2+^ species of MB) using a Varian Cary 300 UV–visible spectrophotometer (Varian, Inc., Palo Alto, CA, USA). The H_2_S concentration of each sample was calculated against a calibration curve of Na_2_S in the range 1–300 μM. The results, produced in triplicate, were plotted as released [H_2_S] *vs* time [58].

### 3.4. In Vitro Antiproliferative Studies

The human triple-negative breast cancer cell line (MDA-MB-231), colon cancer cell line (HCT-116), and lung cancer cell line (A549) used in this study were obtained from the American Type Culture Collection (ATCC). These cell lines were cultured as monolayers in Dulbecco’s Modified Eagle’s Medium (DMEM) supplemented with 10% FBS, 2 mM *l*-glutamine, 100 U/mL penicillin, and 100 µg/mL streptomycin sulfate. For HCT-116 cells, cobalt (II) chloride (CoCl_2_) was employed as an inducer of HIF-1α to create a chemically induced hypoxic environment. The cells were sub-cultured using trypsin/EDTA solution, counted with a hemocytometer, and plated on 96-well plates (5000 cells/well), allowing them to form a semi-confluent monolayer overnight. Subsequently, cell monolayers were treated in quadruplicates with either the vehicle (DMSO, 0.1% *v*/*v*), test samples (**22**, **23**, **26**, **29**, and **30**), or Staurosporine as a positive control, with an exposure time of 72 h. After the exposure period, MTT solution in PBS (5 mg/mL) was added to all wells, including a no-cell blank, and left to incubate for 90 min. The formation of formazan crystals was visually confirmed using phase-contrast microscopy. DMSO (100 µL/well) was then added to dissolve the formazan crystals with shaking for 10 min, after which the absorb absorbance was read at 590 nm against no-cell blanks using a FLuo Star Optima microplate reader (BMG technologies, Ortenberg, Germany). Cell proliferation was calculated by comparing the OD values of the DMSO control wells with those of the samples, represented as % proliferation relative to the control. A dose–response experiment was conducted on samples that produced ≥50% loss of cell proliferation using five serial 2-fold dilutions (50, 25, 12.5, 6.25, and 3.125 µM) of the sample. IC_50_ values (concentration of the sample causing 50% loss of cell proliferation compared to the vehicle control) were calculated using non-linear regression curve fitting on GraphPad Prism V.6.0 software [78].

### 3.5. Cell Cycle Analysis

HCT-116 (colon cancer) and A549 (lung cancer) cell lines underwent a 24-h treatment with compounds **23**, **26**, and **30** at their respective IC50 concentrations. Following treatment, cells were washed twice with ice-cold phosphate-buffered saline (PBS). Subsequently, the treated cells were harvested through centrifugation, fixed in ice-cold 70% (*v*/*v*) ethanol, washed with PBS, re-suspended in 100 μg/mL RNase, stained with 40 μg/mL propidium iodide (PI), and subjected to flow cytometry analysis using FACS Calibur (Becton Dickinson, Franklin Lakes, NJ, USA). The cell cycle distributions were calculated using CellQuest software 5.1 (Becton Dickinson) [79].

### 3.6. Annexin V-FITC/PI Dual Staining Assay

Phosphatidylserine externalization was evaluated using the Annexin V-FITC/PI apoptosis detection kit (BD Biosciences, USA), following the manufacturer’s guidelines. Colon cancer (HCT-116) and lung cancer (A549) cell lines were cultured to form a monolayer, after which they were treated with compounds **23**, **26**, and **30** at their IC_50_ concentrations In brief, the cells were harvested through trypsinization, washed twice in PBS, and resuspended in a binding buffer. Subsequently, the cells were re-suspended in 100 μL of binding buffer with the addition of 1 μL of FITC-Annexin V, followed by a 30-min incubation at 4 °C. After rinsing in binding buffer, the cells were resuspended in 150 μL of binding buffer with the addition of 1 μL of DAPI (1 μg/μL in PBS). Flow cytometry analysis was performed using the BD FACS Canto II flow cytometer and the results were interpreted with FlowJo7.6.4 software (Tree Star, Ashland, USA) [79]. 

### 3.7. Statistical Analysis

All statistical analyses were conducted using Prism 9 (GraphPad Software, USA) in demo mode. Data are expressed as means ± standard deviations (SDs) based on a minimum of three independent experiments. Differences between groups were evaluated using one-way analysis of variance (ANOVA), followed by Tukey’s post hoc test for multiple comparisons. Statistical significance was set at *p* < 0.05. IC_50_ values were determined through non-linear regression analysis (curve fitting) to calculate the concentration of compounds needed to achieve 50% inhibition of cell proliferation.

## 4. Conclusions

The hydrogen sulfide gasotransmitter plays a pivotal role in various physio-pathological processes, and its higher but non-toxic therapeutic concentrations, released by H_2_S donors, have demonstrated cytotoxicity against various tumors. Conversely, tumor-associated CAs, specifically IX and XII—and potentially membrane-bound CA IV in cerebral tumors—are implicated in tumor growth, malignant progression, immune system non-responsiveness, and resistance to chemotherapy and radiotherapy. In particular, these CAs are overexpressed in hypoxic conditions, although their expression is also induced by lactate in glycolytic normoxic tumors.

In this study, we propose, for the first time, the application of designed H_2_S donor–CAI derivatives as antitumor agents. These compounds offer a novel approach for treating H_2_S-sensitive CA IX/XII-overexpressing tumors. Hydrogen sulfide exerts its effects on the respiratory chain and oxidative phosphorylation, making it toxic to tumor cells with oxygen-dependent metabolism (normoxic glycolytic or non-glycolytic tumors). It induces oxidative stress, acidosis, cell cycle arrest in G_2_ and M phases, and apoptosis.

On the other hand, CAs play a crucial role in buffering metabolic acidosis caused by the Warburg effect in both hypoxic and glycolytic normoxic tumors. In hypoxia, where extracellular acidification is more pronounced, CA IX and XII act as the primary machinery producing bicarbonate, replenishing various metabolic pathways crucial for cell replication. Thus, dual-action derivatives might be effective in both early-stage and advanced metastatic solid tumors, as shown in Phase II clinical studies of the CA inhibitor SLC-0111. The H_2_S-releasing moiety, being active in cellular mechanisms present in both hypoxic and normoxic conditions, allows the hybrid compound to target a broader range of tumor cells. This versatility enables a more consistent antitumor effect across the entire tumor mass, which often contains regions with varying levels of hypoxia.

Among the synthesized hybrids **19**–**33**, which demonstrate a good inhibition profile in vitro against CA IV, IX, and XII, compounds **22**, **23**, **26, 29**, and **30** emerged as potent and selective inhibitors of hCA IX (K_I_s = 19.1, 40.3, 4.1, 46.2, and 2.4 nM; hCA II/hCA IX = 192, 120, 719, 209, and 286) and XII (K_I_s = 12.9, 8.8, 7.7, 8.4, and 7.0 nM; hCA II/hCA XII = 284, 548, 383, 1148, and 299), while derivatives **22**, **29**, and **30** selectively inhibit CA IV (K_I_s = 46.2, 430.1, and 244.2 nM; hCA II/hCA IV = 104, 29.3, and 18.3). Furthermore, through a spectrophotometric methylene blue method, compounds **22**, **23**, **26**, **29**, and **30** consistently exhibited the release of H_2_S.

The study also investigated the antiproliferative activity of these compounds against three cancer cell lines (MDA-MB-231, HCT-116, and A549) using the MTT assay. Under normoxic conditions, the compounds exhibited significant antiproliferative effects, with HCT-116 being the most sensitive, followed by MDA-MB-231 and A549. Compound **23** displayed superior cytotoxicity across all cell lines. Under chemically induced hypoxia, derivative **30** demonstrated a 2-fold increase in cytotoxicity (0.93 ± 0.02 µM), indicating an activity related to tumor-associated CA inhibition.

The impact of highly cytotoxic compounds **23**, **26**, and **30** on the cell cycle of the most inhibited cell lines (HCT-116 and A549) was evaluated using DNA flow cytometric analysis, highlighting a disruption characterized by a reduction in the percentage of cells in G_0_/G_1_ and S phases, accompanied by a notable increase in the population in G_2_/M and Sub-G_1_ phases. This was reinforced by an Annexin V-FITC/PI double staining (AV/PI) apoptosis assay, showing that the increased sub-G_1_ population in treated HCT-116 and A549 cells with **23**, **26**, and **30** are correlated with induced apoptosis, with respective fold increases of 37.2, 24.5, and 32.9 against HCT-116 cells and 14.2, 13.1, and 19.9 against A549 cells compared to untreated cells.

These promising findings underscore the potential of H_2_S releaser-CAI compounds as effective tools in tumor treatment. Furthermore, as demonstrated in our prior research, these compounds exhibit efficiency in reducing inflammation, making them versatile agents capable of addressing both cancer and the associated inflammatory states on multiple fronts.

## Figures and Tables

**Table 1:** Inhibition constants (K_I_) of H_2_S releaser-CAI derivatives **19**–**33** against human CA isoforms I, II, IV, IX, and XII using the standard sulfonamide inhibitor acetazolamide (AAZ) by a stopped-flow CO_2_ hydrase assay [59].

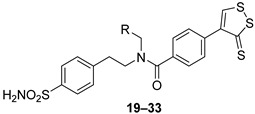						
		K_I_ (nM) ^a,b^				
Cmpd	R	hCA I	hCA II	hCA IV	hCA IX	hCA XII
**19**	-C_6_H_5_	770.7	479.9	4339	43.0	9.7
**20**	-4-F-C_6_H_4_	5324	599.8	>10,000	46.3	65.3
**21**	-4-Cl-C_6_H_4_	5505	2974	430.1	24.0	9.8
**22**	-4-Br-C_6_H_4_	7704	3661	1042	19.1	12.9
**23**	-4-CN-C_6_H_4_	>10,000	4826	46.2	40.3	8.8
**24**	-4-N(CH_3_)_2_-C_6_H_4_	4261	995.4	3412	22.7	9.5
**25**	-4-NO_2_-C_6_H_4_	7453	918.7	>10,000	36.6	32.9
**26**	-2-OCH_3_,4-NO_2_-C_6_H_3_	6697	2950	4093	4.1	7.7
**27**	-4-OCH_3_-C_6_H_4_	2215	589.1	1586	50.3	76.4
**28**	-3,4-diOCH_3_-C_6_H_3_	890.3	218.5	292.6	18.6	8.9
**29**	-4-SCH_3_-C_6_H_4_	7210	9646	403.8	46.2	8.4
**30**	-1-naphthyl	>10,000	4464	244.2	15.6	7.0
**31**	-benzo[b]thiophen-3-yl	>10,000	2482	48.0	2.4	8.0
**32**	-CH_2_C_6_H_4_	4387	660.9	243.2	125.7	9.6
**33**	-CH_2_CN	429.2	60.4	523.1	28.7	9.1
**AAZ**	-	250.0	12.5	74	25.0	5.7

a. Mean from three different assays, by a stopped-flow technique (errors were in the range of ± 5−10% of the reported values). b. K_I_ values from Ref. [58].

**Table 2:** H_2_S-releasing C_max_ and half-lives (t/2) of compounds **3**, **22**, **23**, **26**, **29**, and **30**.

Cmpd	C_max_	t/2 (min)
**3 ^a^**	12.1	3.1
**22**	8.0	4.7
**23**	8.2	5.2
**26 ^a^**	7.9	4.9
**29 ^a^**	8.2	4.6
**30 ^a^**	8.0	5.0

a Data from ref. [58].

**Table 3:** In vitro anti-proliferative activity evaluation of derivatives **22**, **23**, **26**, **29**, and **30** against MDA-MB-231, HCT-116, and A549 cancer cell lines under normoxic conditions, using Staurosporine as a standard.

Cmpds	IC_50_ (µM) ^a^		
	MDA-MB-231	HCT-116	A549
**22**	6.31 ± 0.22	6.62 ± 0.21	23.72 ± 1.61
**23**	1.53 ± 0.04	1.24 ± 0.04	4.53 ± 0.12
**26**	10.43 ± 0.61	2.30 ± 0.072	7.47 ± 0.31
**29**	3.25 ± 0.09	10.13 ± 0.66	60.91 ± 2.17
**30**	9.59 ± 0.31	2.30 ± 0.14	1.78 ± 0.04
**Staurosporine**	8.24 ± 0.34	10.13 ± 0.35	5.11 ± 0.19

**^a^** IC_50_ values are the mean ± S.D. of three separate experiments.

**Table 4:** In vitro anti-proliferative activities of **22**, **23**, **26**, **29**, and **30** against HCT-116 cancer cell line under chemically induced hypoxic conditions with CoCl_2_·6H_2_O, using Staurosporine as standard, and the calculated ratio of IC_50_ normoxia/IC_50_ hypoxia.

Cmpds	IC_50_ (µM) ^a^		IC_50_ Normoxia / IC_50_ Hypoxia
	HCT-116 (Hypoxia)	HCT-116 (Normoxia)	
**22**	11.38 ± 0.22	6.62 ± 0.21	0.6
**23**	4.27 ± 0.08	1.24 ± 0.04	0.3
**26**	3.73 ± 0.07	2.30 ± 0.072	0.6
**29**	12.66 ± 0.25	10.13 ± 0.66	0.8
**30**	0.93 ± 0.02	2.30 ± 0.14	2.5
**Staurosporine**	11.75 ± 0.23	10.13 ± 0.35	0.8

^a^ IC_50_ values are the mean ± S.D. of three separate experiments.

**Table 5:** Effects of compounds **23**, **26**, and **30** on the phases of the HCT-116 and A549 cell cycle.

	Cmpds	%G0-G1	%S	%G2/M	%Sub-G1
**HCT-166**	23	20.35	23.29	32.2	24.16
	26	27.14	24.13	30.29	18.44
	30	28.22	18.4	31.39	21.99
	Control	49.16	38.6	10.8	1.44
**A549**	23	29.65	26.28	28.09	15.98
	26	29.49	22.54	33.05	14.92
	30	22.93	20.77	34.96	21.34
	Control	56.09	35.55	6.36	2

**Table 6:** Distribution of apoptotic cells in the Annexin V-FITC/PI dual staining assay in HCT-116 and A549 cells after treatment with **23**, **26**, and **30**.

	Cmpds	Early Apoptosis (Lower Right %)	Late Apoptosis (Upper Right %)	Total
**HCT-166**	**23**	7.69	21.33	29.02
	**26**	4.13	14.96	19.09
	**30**	10.23	15.49	25.72
	**Control**	0.47	0.31	0.78
**A549**	**23**	6.91	10.13	17.04
	**26**	6.47	9.3	15.77
	**30**	5.36	18.59	23.95
	**Control**	0.89	0.31	1.2

## Data Availability

The datasets and materials used and/or analyzed during the current study are available from the corresponding author upon reasonable request.
